# Sero-prevalence, risk factor and force of infection estimates for foot-and-mouth disease in two cattle rearing areas of Cameroon

**DOI:** 10.3389/fvets.2026.1959956

**Published:** 2026-10-22

**Authors:** Barend M.deC. Bronsvoort, Georgina Limon, Lucy Ndip, Vincent N. Tanya, Kenton L. Morgan, Victor Ngu Ngwa, Charles K. Nfon, Robert F. Kelly, Bram A. D. van Bunnik, Lina González Gordon

**Affiliations:** 1 Epidemiology, Economics and Risk Assessment (EERA) Group, The Roslin Institute, Royal (Dick) School of Veterinary Studies, University of Edinburgh, Midlothian, United Kingdom; 2 The Pirbright Institute, Surrey, United Kingdom; 3 Laboratory of Emerging Infectious Diseases, University of Buea, Buea, Cameroon; 4 Cameroon Academy of Sciences, Yaoundé, Cameroon; 5 Institute of Veterinary Sclence, University of Liverpool, Liverpool, United Kingdom; 6 School of Veterinary Medicine and Sciences, University of Ngaoundere, Ngaoundere, Cameroon; 7 National Centre for Foreign Animal Disease, Winnipeg, MB, Canada

**Keywords:** Cameroon, cattle, FMD, foot-and-mouth disease, force of infection, R_0_, reproductive number

## Abstract

Foot-and-mouth disease (FMD) continues to be a major global disease, directly impacting endemic regions and indirectly affecting FMD-free regions. Reliable estimates of key epidemiological parameters, such as transmission rates, from empirical field data are scarce yet essential for developing statistical and mathematical models to assess control strategies and inform policy. Here, we estimate the seroprevalence and risk factors for FMD across the North-West Region (NWR) and Vina Division (VIN) of Cameroon and use these data to estimate the force of infection and the reproductive numbers, R_0_ and R_t_. A total of 1,498 cattle from 100 herds across the Northwest Region and Vina Division of Cameroon were sampled. Evidence of recent FMD occurrence was widespread, with 60.1% of herdsmen in the NWR and 76.8% in the VIN reporting clinical FMD within the previous 12 months. FMD non-structural protein (NSP) antibody responses increased with age before reaching a plateau, consistent with cumulative exposure in endemic settings. Using an empirically derived ELISA cutoff (>40 PI), the design-adjusted animal-level seroprevalence was 38.2% [95% confidence intervals (CI): 31.3–45.8] in the NWR and 56.7% (95% CI: 47.8–65.2) in the VIN, demonstrating substantial virus circulation in both regions and significantly greater exposure in the VIN. Age-stratified analyses revealed a rapid increase in seroprevalence during the first 4–5 years of life, with force of infection estimates almost two-fold higher in the VIN (*λ* = 0.382) than in the NWR (*λ* = 0.197). Correspondingly, the basic reproduction number (R₀) was estimated at 3.04 (95% CI: 2.84–3.26) in the VIN and 2.05 (95% CI: 1.93–2.18) in the NWR. After accounting for population immunity and test performance, the effective reproductive numbers (Rt) were 1.14 (95% CI: 1.02–1.22) and 1.23 (95% CI: 1.15–1.30), respectively for the VIN and NWR. Foot-and-mouth disease is endemic in both study regions of Cameroon, with evidence of substantial transmission and cumulative exposure occurring early in life. Despite the lack of serotype-specific data, age-stratified serology provided robust estimates of population-level transmission, highlighting marked regional variation, the complexity of estimating epidemiological parameters in endemic settings, and the need to further explore the factors driving local disease dynamics.

## Introduction

Foot-and-mouth disease (FMD), caused by the single-stranded positive-sense RNA foot-and-mouth disease virus (FMDV; *Aphthovirus, Picornaviridae*), is one of the most important livestock diseases globally. There are seven distinct serotypes recognized, known as O, A, C, SAT1, SAT2, SAT3, and Asia1, of which only Asia1 has not been reported in Africa. Serotype C has not been reported anywhere since 2004 (1) and may have become extinct in Africa and South America. Although clinically all FMD serotypes are generally characterized by fever and the appearance of vesicular lesions around the mouth and the coronary band of the hoof, epidemiologically they are better, where possible, considered as separate diseases based on serotypes and strains due to differences in pathogenesis and transmission rates, as well as the lack of cross-reactivity between serotypes and the need for serotype- and strain-specific vaccines (2).

FMD typically causes high morbidity but generally low mortality, except in very young animals, when severe myocarditis can lead to high mortality. However, the impacts are substantial, reflecting both direct and indirect losses from reduced production, changes in herd structure, the costs of FMD control, and poor access to lucrative markets for live livestock, animal products (e.g., meat and dairy), and by-products (e.g., hides, bones, and wool) (3). Estimates for the annual impact of FMD in terms of visible production losses and vaccination in endemic regions alone range from US$6.5 to US$21 billion (3).

Under current WOAH rules, failure to control FMD results in major barriers to international trade. The presence of a large endemic reservoir, primarily across Asia and Africa, combined with poor reporting and surveillance, poses a huge risk to disease-free countries and those trying to control disease with vaccination. This is exemplified by the recent spread of serotype O into Indonesia in 2022 (4) and the incursion of Southern African Territories (SAT) 2 from Africa into the Middle East and Turkey in 2023 (5), as well as, most recently, the outbreaks of serotype O in northern Europe (6–8) and SAT1 in Cyprus and Greece (9). While recent epidemics in traditionally FMD-free countries have not reached the scale of the 2001 UK outbreak, they continue to demonstrate the vulnerability of disease-free regions to transboundary incursions (10, 11).

Although livestock disease control is largely considered a private good, there is a growing recognition that widespread FMD control generates substantial public goods, justifying the need for national and international public investment (10). Strengthening the scientific evidence base, expertise, and capacity of endemic countries to monitor, understand, and control FMD can deliver wider societal benefits while also enhancing the control of other transboundary livestock diseases, as recognized in the global strategy (3, 11). These include positive effects on food security and nutrition, poverty reduction, and widespread and safer global trade in livestock and livestock products. Additional indirect benefits include improved animal health, reduced antibiotic use and misuse, decreased selection pressure for antimicrobial resistance (AMR) (10, 12) and the potential to lower greenhouse gas emission intensities, often associated with high disease burdens and suboptimal productivity (10, 12). However, particularly for smallholders or pastoralists in low- and middle-income (LMIC) settings, the application of controls designed for high-income countries can have more detrimental impacts than the disease itself. For instance, closure of livestock markets without compensation can disrupt livelihoods and create major social tensions and economic hardships (13).

Cameroon is a major livestock-producing country in Central Africa, supplying cattle to markets in Nigeria, Gabon, and Equatorial Guinea, while also satisfying domestic market demands. The country is part of what is known as FMDV pool 5, with distinct sub-lineages of serotypes O, A (IV), SAT1, and SAT2 (VII) (14) co-circulating. Epidemiologically, the broader region occupies a strategically important position, as it sits at the narrow bridge that connects the West Central African pool 5 to the East African pool 4. The extensive livestock movements, particularly from east to west, position the region as a significant mixing vessel for viruses between FDMV pools (14–16). Furthermore, Cameroon also lies at the crossroads of several new trans-Saharan road networks linking Central and North Africa, increasing the risks of virus spread from endemic areas to disease-free zones (17).

Within this complex epidemiological landscape, there is an important opportunity to leverage biobanked samples and historical epidemiological data to estimate disease burden and derive key epidemiological parameters required for disease modeling, such as the force of infection and reproduction numbers (18). Empirical parameter estimates from endemic settings are lacking and are essential to parameterize reliable statistical and mathematical models exploring disease risk and evaluating the potential impact of alternative disease control strategies through scenario modeling in both endemic and epidemic contexts (19).

This study describes the results from a cross-sectional survey using biobanked serum samples from 2013 to screen for non-structural protein (NSP) antibodies in two areas of Cameroon. The main objectives were to estimate the seroprevalence and risk factors for FMD in the two study areas, as well as to estimate the force of infection (FOI), the basic reproduction number (R_0_), and the derived effective reproduction number (R_t_). This information is essential to support evidence-informed decision-making and improve preparedness for future epidemics.

## Materials and methods

In documenting this analysis, the authors have followed the STROBE guidelines for reporting observational epidemiological studies (20).

### Study sites

The study was conducted in two sites in the North-West Region (NWR) and Vina Division (VIN) of the Adamawa Region of Cameroon. Both are of similar geographical size (~17,000 km^2^) yet differ considerably in their landscape, population density, and cattle-keeping practices. The NWR lies within Cameroon’s fertile mountainous highlands, with elevations ranging from approximately 500 to over 3,000 m above sea level. It is densely populated (1,804,695 people), including many non-cattle-keeping groups, while also being home to the regional capital, Bamenda, one of Cameroon’s largest cities. In contrast, the VIN is part of the fertile Guinea savannah plateau in the Adamawa Region and has a substantially smaller human population (317,888 people), dominated by cattle keeping. However, the cattle population is larger in the NWR, with an estimated 506,548 cattle grazing there compared to 176,257 head in the VIN (21). Despite these differences, cattle production in both areas is based largely on extensive pastoralist systems, although patterns of mobility differ considerably. Ethnically, the cattle-keeping populations in the NWR identify as “Mbororo” and are traditionally more nomadic than the more sedentary “Fulbe,” who are the majority in the VIN. Levels of transhumance also vary, with ~44% of herds undertaking transhumance in the NWR compared to only ~6% in the VIN. This is very low compared with the rest of the Adamawa Region and reflects access to feed concentrates in Ngaoundere (VIN regional capital) (21, 22). The choice of sites was driven by the original study on bovine tuberculosis and sampling from the populations supplying the major abattoirs where sampling was also conducted in Bamenda and Ngaoundere (21).

Veterinary services were predominantly provided by the government through the Ministry of Livestock, Fisheries and Industrial Agriculture/Ministere de l’Elevage des Peches et Industries Animales (MINEPIA), with local veterinary technicians stationed at Zootechnical and Veterinary Sanitary Control Centers (ZVSCC) distributed across the country (22). Their responsibilities include registration of local livestock keepers, disease control mainly through annual vaccination campaigns, meat inspection, and regulation of livestock markets and animal movements.

### Study design

A cross-sectional survey was conducted between January and May 2013 in the NWR and between September and November 2013 in the VIN. These participants were pastoralists whose herds were listed in the Ministry of Livestock, Fisheries and Animal Industries vaccination records from 2012 at 81 local veterinary centers in the NWR and 31 in the VIN. A total of 5,053 pastoralist herds in the NWR and 1,927 in the VIN, herd sizes ranging from 1–215 cattle per herd were included in the sampling frame. The list of herds in each site was stratified by administrative area; seven Divisions in the NWR and eight sub-Divisions within the VIN and a random sample of herds was taken from each site proportional to the total number of herds listed in each stratum. This survey was part of a larger study of bovine Tuberculosis (bTB) and liver fluke, and the sample size was based on a clustered random sample of cattle, assuming a cattle-level prevalence of bTB of ~10%, a within-herd variance of 0.15 and between-herd variance of 0.01, an average herd size of 70, a relative cost of 12:1 for herd, and a relative error of ±15% (Survey Toolbox; AusVet) (23). This gave a target sample size of 15 cattle per herd and 88 herds under the simplifying assumption of perfect test performance. Within each herd, animals were stratified into three age classes; >6 months to <2 years old (young), 2–5 years old (adult), and older than 5 years (old), and five animals selected from each stratum. To allow for potential losses or dropouts and to have balanced samples from the two sites, we aimed for 50 herds in each of the two sites, the NWR and VIN.

### NSP FMD ELISA

The NCFAD/NCPanaftosa 3ABC competitive ELISA (3ABC cELISA) was performed in Canada as previously described (24). Briefly, plates were coated with a recombinant FMDV 3ABC antigen. Equal volumes of serum and anti-FMDV 3B monoclonal antibody (mAb) were then added to the plate. Antibodies in the serum samples compete with the anti-FMDV 3B mAb for a specific epitope on the recombinant protein. Horseradish peroxidase (HRP)-conjugated goat anti-mouse IgG (Jackson ImmunoResearch Laboratories Inc.) was then added, followed by 3,3′,5,5′-tetramethylbenzidine dihydrochloride (TMB) substrate (Sigma-Aldrich, St. Louis, MO, USA). After a 15-min incubation at room temperature, the color development was stopped with 2 M sulfuric acid, and the OD450 was determined using a plate reader (Photometer Multiskan reader; Labsystems, Foster, VA, USA). Test sera containing anti-FMDV 3ABC antibodies inhibited binding of the mAb to the recombinant FMDV 3ABC antigen to various degrees, and the results were expressed as the percentage of inhibition (PI). Serum samples with PI ≥ 50% were considered positive for antibodies to FMDV 3ABC. The published parameters for the NCPanaftosa 3ABC competitive ELISA (based on the 50% cutoff) are: ~94% sensitivity (Se) and ~99.3% specificity (Sp) (24). However, following analysis this was adjusted to ≥ 40% (see results).

### Interferon-γ assay and diagnostic tests for other infectious diseases

As part of the risk factor analysis, we were interested in associations with co-infections/exposures, especially infections that may have an immunomodulating effect including bovine viral diarrhoea (BVD), bovine enzootic leukosis (BLV), *Mycobacterium bovis* (bTB), or *M. avium* subspecies paratuberculosis (paraTB). The IFN-γ assay (Prionics Bovigam®) was used to screen for *M. bovis*, and commercial ELISAs (ID. Vet https://www.innovative-diagnostics.com) for the other infections [see Kelly et al. (25) for details of the tests used].

### Descriptive statistics and mapping

All data analyses and mapping were carried out using the R statistical language (26) (version 4.0.4) within RStudio (Version 1.2.1335). Maps were generated using the *ggmap* with *ggplot2* package (27) and the shapefiles obtained from the open-access GADM database of Global Administrative Areas (www.gadm.org).

The population seroprevalence estimates were adjusted for the survey design using animal and herd-level weighting. The animal-level weighting, waj, was calculated as the number of animals sampled in a given herd (na_j_), divided by the herd size (NA_j_), $\frac{na_{j}}{NA_{j}}$ , and a herd-level weighting, whk, was calculated as the number of herds sampled divided by the number of herds in the sampling frame, $\frac{nh_{k}}{NH_{k}}$ , for a given administrative division/sub-division. This gave the overall weighting for an animal of $wa_{j}\times wh_{k}$ . The *survey* package (28) was used to estimate the design adjusted seroprevalences within herds and at administrative level and for the two sites assuming a perfect test [design-adjusted estimate (Daj)]. All estimates are given with 95% confidence intervals (CI).

### Force of infection (*λ*), basic reproduction number (R_0_), and effective reproduction number (R_t_)

The force of infection (FOI), *λ*, is defined as ‘the rate at which susceptible individuals become infected per unit time’, or the probability that a susceptible individual will become infected per unit time, and depends on the number of infectious individuals in a population and their contact rate with susceptible individuals. The FOI, *λ,* was estimated using three different methods, and Akaike’s Information Criteria (AIC) was used to compare the model fits to the data. A catalytic model, first described by Muench, that estimates a constant FOI was compared to two age-dependent models, one in which FOI is a linear function (Griffith’s model), and one in which FOI is a quadratic function of age groups (Grenfell–Anderson model). Generalized linear models were used as a statistical framework for each of the three models, adapted from Hens et al. (29). Data were organized into 1-year-wide age groups, except for animals over 10 years of age, which were all grouped in a 5-year-wide age band.

*R_0_,* the basic reproduction number, was estimated as follows. The average life expectancy can be estimated as $\sum_{x=1}^{\infty }l_{x}$ , where $l_{x}$ is the survival rate at age *x* and is calculated as the ratio of the number of animals at age *x* divided by the number of animals in age class 1 (animals aged up to 1 year old). However, due to the sampling structure, in which five animals from three age classes were selected, this would produce a potential bias, and therefore, a literature-based estimate of 6 years (30) was used, with a sensitivity analysis extending 2 years either side of this value. For an individual of age *a*, the standard SIR (susceptible, infectious, recovered) model predicts that the probability that an individual is still susceptible at age *a*, is given by P(*a*) = exp (− *aμ*(*R*_0_–1)) under some simplifying assumptions, where *a* is the animal’s age and *μ* is the reciprocal of the average life expectancy (31). Since the numbers of the susceptible and infectious animals are binomially distributed, the likelihood function of these numbers can be obtained as a function of R_0_. R_0_ was then inferred as the value that maximized the log-likelihood function:

$$\begin{array}{l} \ell (R_{0})=\sum_{i=0}^{n}\log(\exp(-a_{i}\mu (R_{0}-1)))\\ +\sum_{i=0}^{m}\log(1-\exp(-b_{i}\mu (R_{0}-1))) \end{array}$$

where *n* is the number of susceptible animals (sero-negative) of age *a*_1_, …, *a_n_*, and *m* is the number of seropositive animals (ages *b*_1_, …, *b_m_*) for all farms (32).

R_t_, the effective reproduction number in this population, can be estimated from R_0_ by taking into account the seroprevalence estimates and the NSP test specificity (Sp) and sensitivity (Se). The estimated Se and Sp after shifting the cut-off to 40% were 97.3 and 98.1%, respectively (see Supplementary Appendix A). Mean true seroprevalence values were calculated per study site and adjusted for Sp and Se of the NSP test using the 40% cut-off (design- and test-adjusted estimate [DAT]) as follows:

$$p_{\mathrm{obs}}=\frac{\mathrm{no}.\text{positive tests}}{\text{total tests}},$$

After which the adjusted prevalence was calculated as:

$$p_{\text{true}}=\frac{p_{\mathrm{obs}}+\mathrm{sp}-1}{\mathrm{se}+\mathrm{sp}-1}$$

R_t_ can then be calculated using $R_{t}=R_{0}(1-p_{\text{true}})$ .

### Mixed effects logistic regression modeling

At the time of sampling the herds, a questionnaire was also administered to each herdsman, as described in Kelly et al. (21). Several variables from the questionnaire were identified as being of interest for the risk of FMD seropositivity. This included age, breed, and factors influencing contact rates such as recent livestock purchases, contact with livestock kept at the homestead (sheep and goats), and contact with wildlife during transhumance (antelopes and buffalo). Co-infection status data available for bovine viral diarrhoea (BVD), enzootic bovine leucosis (EBL) and paratuberculosis (paraTB) was also included. A conceptual graphical model was developed to identify important variables in terms of their biological plausibility as risk factors for FMD exposure or as potential sources of confounding. Based on this, a univariable analysis was conducted, and all variables were assessed for multi-collinearity to avoid issues with multivariable regression model estimation. Variable selection was performed using a backward stepwise approach. A multivariable mixed-effects logistic regression models was fitted using the *glmer* function of package *lme4* (33) with the individual-level NSP antibody result as the binary outcome. The Akaike’s Information Criterion (AIC) was used to select the best-fitting model, and model fit was assessed using the *performance* package (34).

## Results

A total of 1,498 animals were sampled from 50 herds in the NWR and 50 herds in the VIN of the Adamawa Region. Herdsmen in Cameroon were reported to be good at recognising the signs of FMD (35) and 60.1% (DAj 95% CI: 45.9–73.0) reported having animals with FMD in the last 12 months in the NWR compared with 76.8% (DAj 95% CI: 62.4–87.0) in the VIN. Interestingly, 32.9% (DAj 95% CI: 21.0–47.0) of herdsmen reported deaths they attributed to FMD in the NWR compared to only 17.1% (DAj 95% CI: 8.6–31.0) in the VIN possibly reflecting different serotypes or strains with different epidemiological characteristics.

### FMD-NSP antibody patterns

For both study sites the percentage inhibition (PI) of the ELISA readout increased with age up to ~4–5 years of age and then remains relatively constant (Figure 1a). However, the VIN appears to have consistently higher mean PI across all ages, suggesting higher levels of exposure and virus circulation. The distribution of PI values is plotted in Figure 1b and shows a bimodal pattern with the seropositive animals on the right. The manufacturer’s cutoff was ≥50 PI, but the distribution observed here suggested that ≥40 may be more appropriate and would move 36 animals from negative to positive, potentially increasing the Se at the expense of the Sp [Se_40_ = 97.3 (92.9–99.8); Sp_40_ = 98.1 (95.2–99.9)]. For all subsequent analyses, the ≥40 PI cut-off was used. Figure 1c plots each animal clustered in a column by herd and ordered by herd seroprevalence.

**Figure 1:**
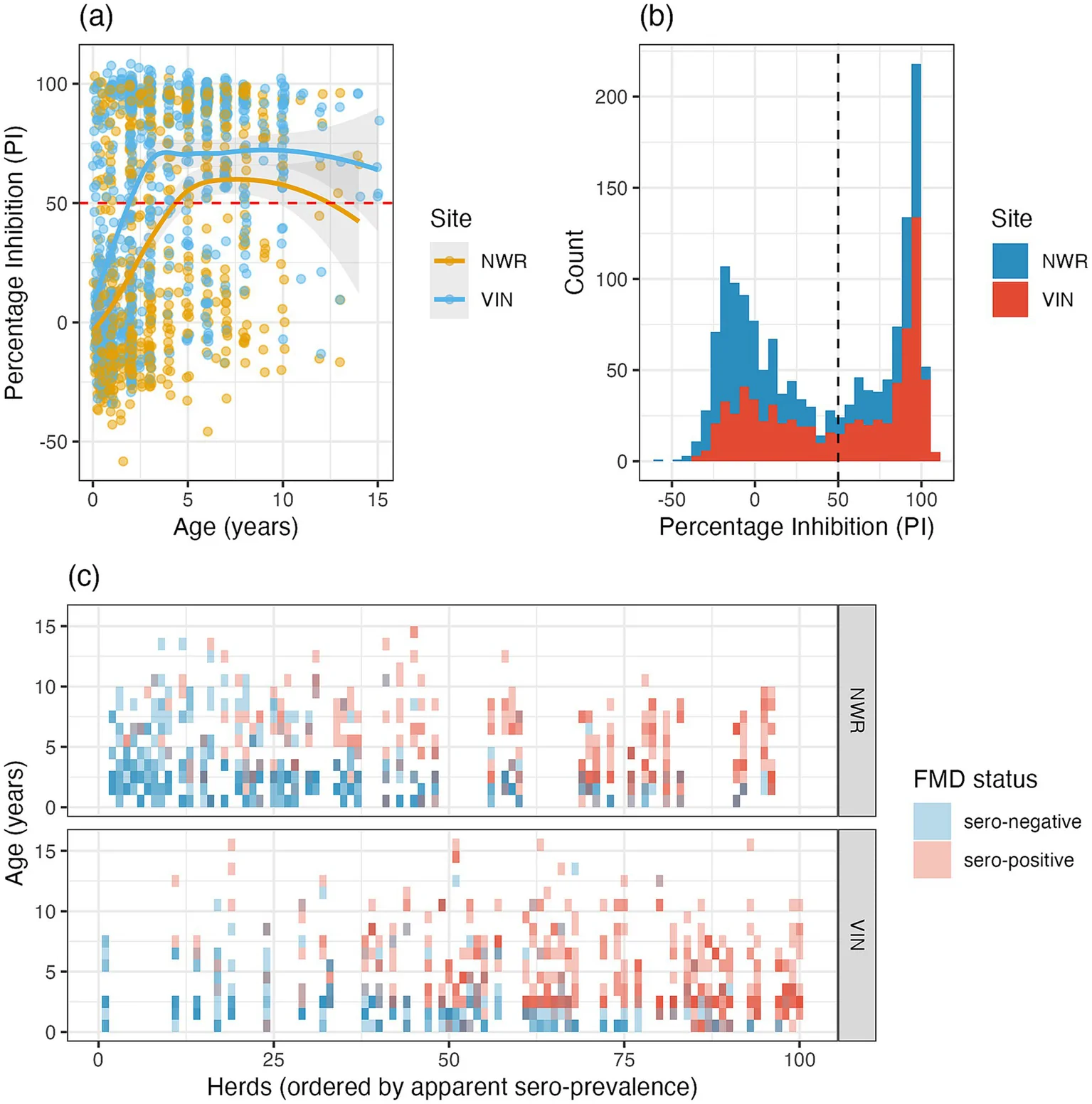
**(a)** Raw individual animal (*n* = 1,498) percentage inhibition (PI) of the FMD NSP ELISA (ID.VET) plotted by age and stratified by study site in Cameroon. The dashed line is the cut off above which animals would be classified as positive. **(b)** Stacked histogram of individual animal PI stratified by site. **(c)** Tile plot of individual animal seropositivity status, where seronegative animals are represented by blue tile and seropositive animals by red tile (binary), by age in years (y-axis), grouped by herd (i.e., all the 15 animals from a herd are represented in the same vertical column), ordered by herd prevalence with lowest on the left to highest on the right (x-axis), and stratified by study site. Variation in color opacity arises when tiles representing more than one animal are overlaid.

### Seroprevalence of FMD-NSP antibodies

The overall design adjusted animal-level seroprevalence of FMDV antibodies was 38.2% (DAjCI: 31.3–45.8) in the NWR compared to 56.7% (DAjCI: 47.8–65.2) in the VIN. These estimates were consistent with the herd-level reports from herdsmen above.

The study design adjusted animal-level seroprevalences are given in Table 1 and mapped for the two sites in Figure 2. Bui and Ngoketunjia in the NWR and most of the sub-divisions in the VIN had high FMD-NSP seroprevalences, with more than 50% of animals being seropositive. As with the raw PIs, the VIN overall had a higher FMD-NSP seroprevalence than the NWR at the time of sampling.

**Table 1:** Design-adjusted (DAj) animal-level seroprevalence (not adjusted for test performance) of FMD-NSP antibodies in cattle in Cameroon in 2013, stratified by division (NWR) and sub-division (VIN).

Division/sub-division	Pos	Total	Design-adjusted seroprevalence	Design-adjusted 95% CI
Northwest region				
Boyo	33	90	0.37	0.15–0.67
Bui	101	195	0.51	0.36–0.71
Donga Mantung	51	180	0.26	0.15–0.39
Menchum	15	75	0.20	0.06–0.48
Mezam	44	105	0.38	0.21–0.58
Mono	18	60	0.32	0.03–0.86
Ngoketunjia	35	45	0.78	0.67–0.86
Vina division				
Belel	96	150	0.64	0.51–0.75
Martap	151	255	0.58	0.50–0.65
Mbé	23	30	0.80	0.00–1.00^*^
Ngan-ha	36	73	0.37	0.08–0.81
Ngaoundere	41	60	0.79	0.23–0.98
Nyambaka	96	180	0.51	0.32–0.71

^*^The small within strata sample size resulted in a high level of uncertainty.

**Figure 2:**
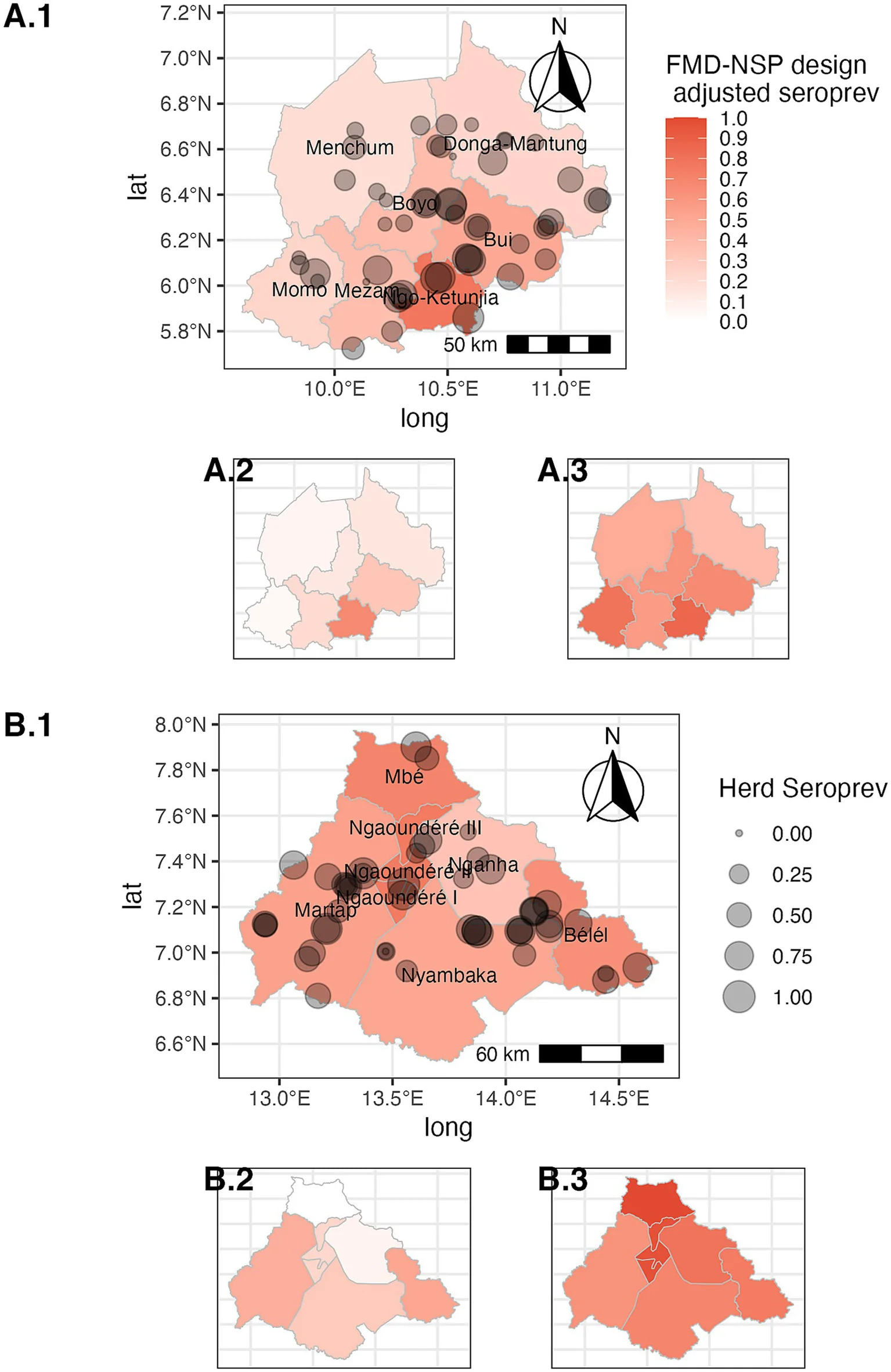
Choropleth maps of the Northwest Region (NWR) **(a)** and Vina Division (VIN) **(b)** in Cameroon, colored according to design-adjusted (DAj) FMD-NSP apparent seroprevalence for the administrative strata, overlaid with the approximate location of individual herds sized by the raw proportion of animals positive within each herd. The smaller inset choropleth maps are for the lower (X.2) and upper (X.3) 95% confidence intervals, respectively, for each site.

### Age-stratified seroprevalence, force of infection, R_0,_ and R_t_

The age-stratified FMD-NSP seroprevalences are plotted in Figure 3 and show an initial rapid increase in seroprevalence with age to around 4–5 years, after which the seroprevalence appears to remain constant. Antibodies to FMD-NSP can vary considerably in duration but have been shown to consistently last for several years even in repeatedly vaccinated animals (36). The FOI, *λ*, was estimated using the three different models and plotted (Figure 3) with their AICs reported in Table 2. Assuming a constant force (catalytic model) it is clear that *λ* is almost double in the VIN (0.382) that in the NWR (0.197), resulting in the higher overall seroprevalences observed. The best fitting model in both study sites was the quadratic model (although only marginally better than the linear models) suggesting that the FOI declines with age in endemic settings. This may reflect different contact patterns with age, with more mixing and transmission in younger animals through movements, sales and transhumance (seasonal migration).

**Figure 3:**
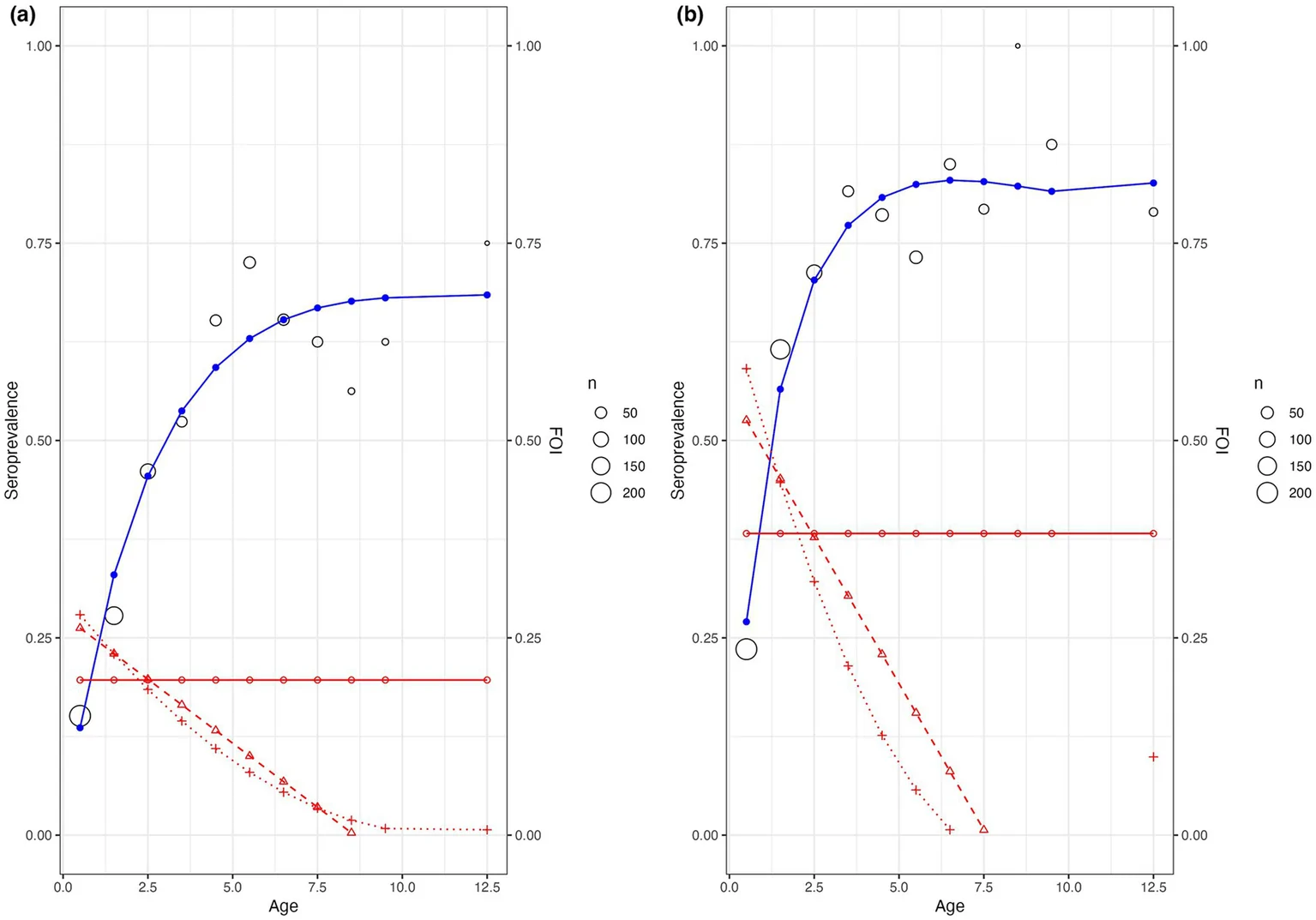
Age-stratified seroprevalence of FMD-NSP antibodies in cattle in two sites **(a)** NWR and **(b)** VIN in Cameroon in 2013. The predicted seroprevalence based on a simple age quadratic function (blue) and the force of infection (λ) based on the Muench/catalytic model (red circle/solid line), the Griffth’s/linear model (red triangle/dashed line), and the Grenfell-Anderson/quadratic model (red cross/dotted line). The black circles show the mean seroprevalence for that age stratum, with the size proportional to the number of animals in that age stratum.

**Table 2:** Force of infection model comparisons for the VIN and NWR populations as plotted in Figure 3.

Model (degrees freedom)	AIC - NWR	AIC - VIN
Muench model – constant FOI (1)	77.71607	119.40451
Griffth’s model – linear FOI with age (2)	57.41134	61.76093
Grenfell Anderson model- quadratic FOI with age (3)	58.44336	57.57431

Columns VIN and NWR contain the Akaike information criteria value (AIC) of model fit.

Based on the average life expectancy of 6 years, R_0_, the average number of new infections an infected animal generates in a naïve population, was estimated to be 2.05 (95% CI:1.93–2.18) for the NWR and 3.04 (95% CI:2.84–3.26) for the VIN, respectively. We included a life expectancy of 6 ± 2 years as a sensitivity analysis and the results are given in Table 3 showing a linear increase in R_0_ with life expectancy. After adjusting for population immunity and test performance, the corresponding effective reproductive numbers (R_t_) estimates were much lower at 1.23 (95% CI: 1.15–1.30) in the NWR and 1.14 (95% CI: 1.02–1.22) in the VIN. Results are plotted across the range of plausible life expectancies in Figure 4 and Supplementary Appendix B.

**Table 3:** Estimates of R_0_ for each of the two study sites over a plausible range of average life expectancies for cattle in the pastoral system in Cameroon.

Site	Life Expectancy	R_0_ (95% CI)	Lower bound	Upper bound
VIN	4	2.36	2.22	2.51
NWR	4	1.70	1.62	1.79
VIN	5	2.70	2.53	2.88
NWR	5	1.88	1.78	1.98
VIN	6	3.04	2.84	3.26
NWR	6	2.05	1.93	2.18
VIN	7	3.38	3.14	3.64
NWR	7	2.23	2.09	2.38
VIN	8	3.72	3.45	4.02
NWR	8	2.40	2.24	2.57

**Figure 4:**
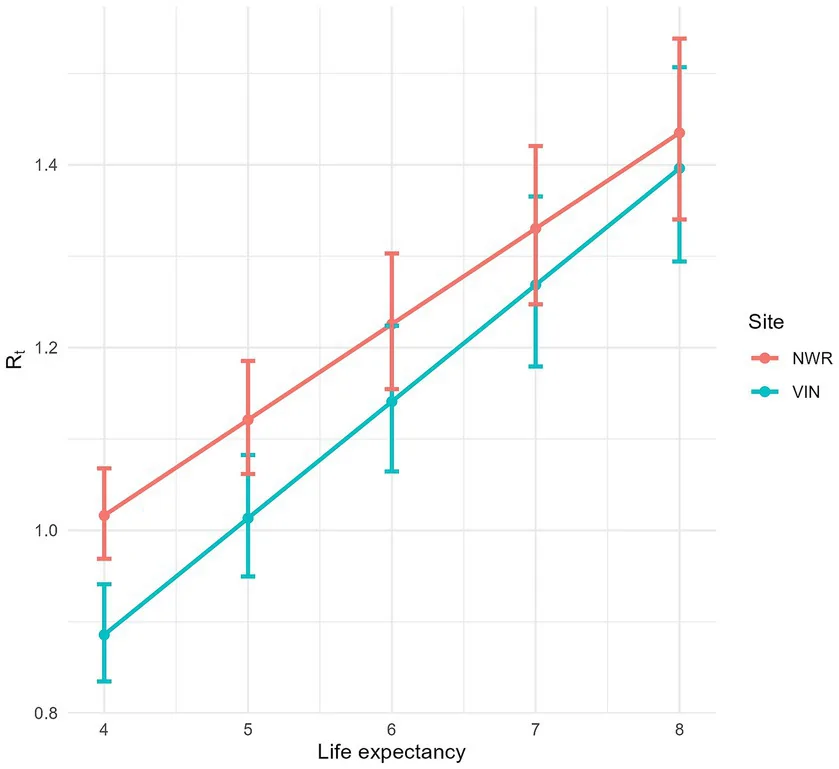
Sensitivity analysis of R_t_ to life expectancy estimates (years) of cattle in the NWR and VIN sites in Cameroon.

### Risk factor analysis

A directed a cyclical graph was developed based on our understanding of likely causal links and pathways between variables and potential confounders included in the original herdsmen questionnaire (Figure 5); all variables are listed in Table 4 along with their association with the FMD serostatus. BVD, EBL, bTB, and para-TB serostatus were included as potential sources of non-specific reactivity in the FMD-NSP assay or potential immune modulators that might impact susceptibility to FMD, rather than as true biological determinants of FMD exposure, with age included as their common upstream determinant given cumulative exposure risk over an animal’s lifetime. The final multivariable logistic regression model showed that age, BVD serostatus, and region were significantly associated with FMD risk (Table 5). Compared with young animals, the odds of FMD seropositivity increased markedly with age (adult: OR = 9.6; old: OR = 34.3), and were also higher in animals from the Vina region (OR = 3.45) and those seropositive for BVD (OR = 1.54).

**Figure 5:**
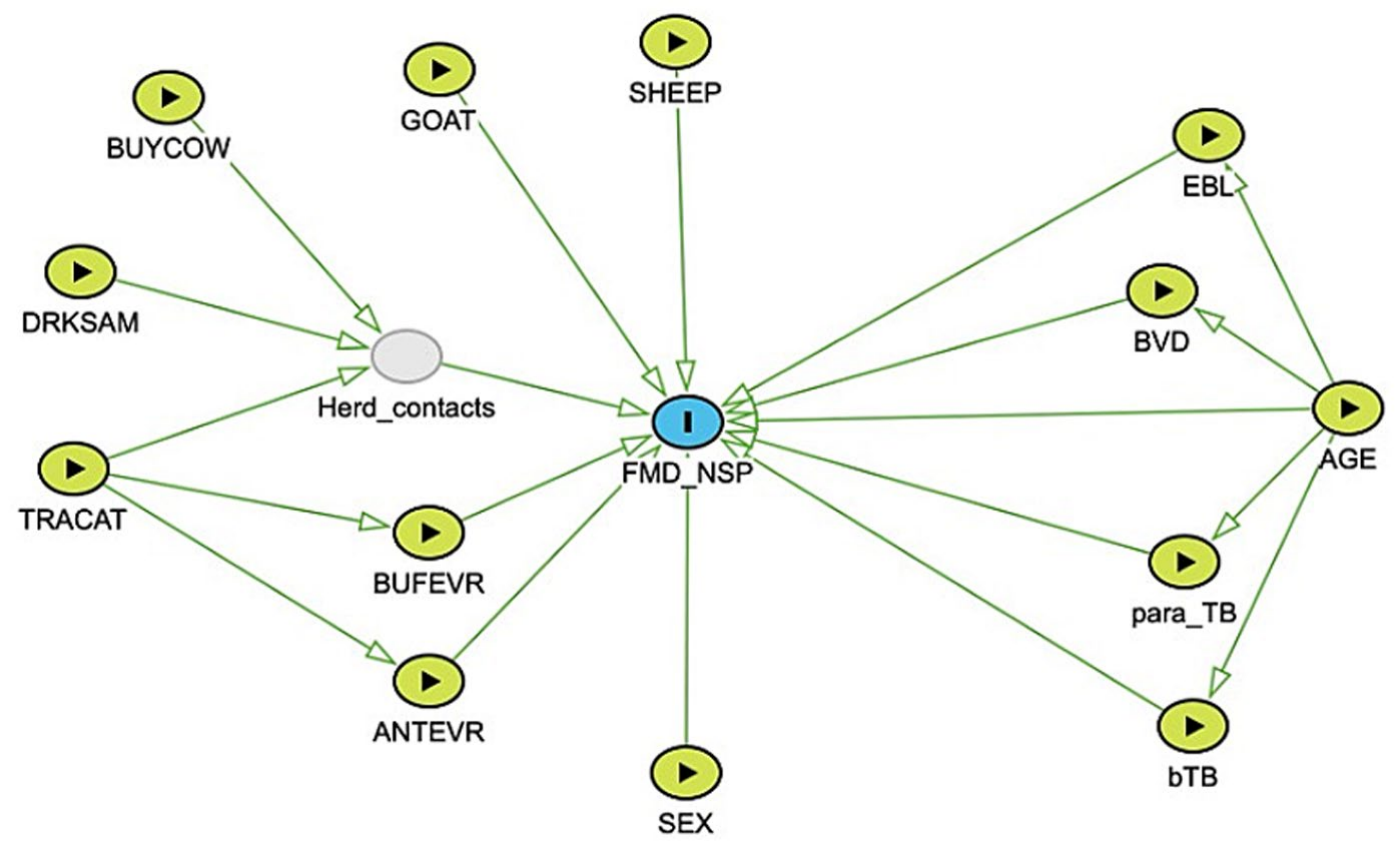
Directed Acyclic Graph illustrating the hypothesized relationships between potential risk factors (listed in Table 3) and FMD animal-level serostatus.

**Table 4:** Univariable analysis of variables of interest in the causal pathway for FMD animal-level seropositivity.

Variable	Label	Levels	FMD pos	FMD neg	OR	95% CI*
Which strata was the farm in/study site	Strata1	NWR VIN	297 443	453 305	1 2.21	1.79–2.74
What other animals, other than cattle, do you currently keep/ rear at your homestead?	SHEEP	N Y	474 266	455 303	1 0.84	0.68–1.04
What other animals, other than cattle, do you currently keep/ rear at your homestead?	GOAT	N Y	522 218	542 216	1 1.05	0.83–1.32
Have you bought any cattle in the past 12 months?	BUYCOW	N Y	366 374	443 315	1 1.43	1.17–1.77
How many other herds use the same watering points as your cattle?	DRKSAM	0 1–5 6+	22 483 235	113 461 184	1 5.37 6.54	3.31–9.08 3.93–11.3
Did any of the cattle presented go on transhumance in the past 12 months?	TRACAT	N Y	572 168	552 206	1 0.79	0.62–1.00
Do any of these cattle presented come in contact with the following wild animals whilst grazing or on transhumance – antelope?	ANTEVR	N Y	357 127	347 187	1 0.66	0.50–0.86
Do any of these cattle presented come in contact with the following wild animals whilst grazing or on transhumance – buffalo?	BUFEVR	N Y	708 32	685 73	1 0.42	0.27–0.66
What is the sex of the animal	SEX	M F	152 588	280 478	1 0.44	0.35–0.56
Which age category is the animal classified as?	AGE	Y (0.5 < 2y) A (2-5y) O (>5y)	103 299 338	398 237 123	1 4.87 10.6	3.70–6.42 7.87–14.3
Was the animal seropositive for BVD?	BVD	N Y	366 374	547 211	1 2.64	2.12–3.30
Was the animal seropositive for EBL?	EBL	N Y	664 76	695 63	1 1.26	0.88–1.82
Was the animal IFNγ positive for bTB?	bTB	N Y	619 63	607 72	1 0.87	0.60–1.26
Was the animal seropositive for para-TB?	para_TB	N Y	704 36	737 21	1 1.79	1.01–3.27

*95% CI are not adjusted for the herd-level clustering.

**Table 5:** Final multi-variable mixed-effects logistic regression model for FMD NSP seropositivity in cattle in Cameroon with herd as the random effect.

Variable	Levels	OR	95%CI
Age category	Y A O	1 9.6 34.3	6.53–14.1 21.3–55.1
BVD status	Seronegative Seropositive	1 1.54	1.07–2.23
Region	NWR VIN	1 3.45	1.65–7.20

## Discussion

Foot-and-mouth disease (FMD) remains a major constraint to livestock production across sub-Saharan Africa, where endemic transmission results in substantial economic losses through reduced productivity, restrictions on livestock trade, and the costs associated with disease management and control. Improving our understanding of FMD dynamics in endemic settings is important for explaining how strains can seemingly disappear for decades before re-emerging, characterising overall transmissibility of the virus and of individual strains and serotypes, and ultimately improving predictions of its spread beyond Africa, as illustrated by the recent spread of SAT1 through the Middle East and into Europe. These epidemiological parameters can only come from field studies and effective surveillance in the countries where the virus is circulating.

The descriptive results presented here are consistent with the persistence of NSP antibodies following natural infection and likely repeated exposures and suggest that most cattle in both study areas had been exposed to FMD viruses at least once during their lifetime. Historically, serotypes O, A, SAT1 and SAT2 have been reported in Cameroon, although data covering this specific period are not available (14, 15). NSP antibody levels following natural infection may remain elevated for at least 3 years (36). They may also remain high over time due to repeated exposure to different serotypes and/or lineages. Neutralising antibody titres wane over time, increasing susceptibility to reinfection with the homologous serotype; heterologous serotypes may cause infection regardless of titre, given the limited cross-reactivity between serotypes (37). While FMDV NSP seropositivity is considered a reliable indicator of previous infection, assessing the timeline of infection or IF the antibody titres reflect single or repeated exposures was not possible in this study. However, from samples collected in the previous year, in 2012, we know that serotype A was circulating in the NWR and SAT2 in the VIN (38). The rapid increase in seroprevalence during the first 4–5 years of age, followed by a plateau, is characteristic of endemic infections in which susceptible animals are progressively infected until the majority of the population has been exposed. The consistently higher seroprevalence observed in the Vina Division (VIN) compared with the Northwest Region (NWR) indicates a higher intensity of transmission and greater cumulative exposure in the Adamawa region or potentially lower turnover rates.

Several studies have recently estimated FOI and R_0_ in Cameroon. Pomeroy et al. (39) estimated lifelong immunity for most serotypes. In our study we do not know the serotypes based on serotype-specific serology as we have only NSP antibodies, but the approach used by Pomeroy et al. (39) approach supports our use of the catalytic model. The estimated basic reproductive number (R₀) was approximately 2.0 in the NWR and 3.0 in the VIN, reflecting the marked differences in seroprevalence and FOI between the two regions. The higher transmission intensity in VIN is notable given its lower apparent livestock density compared with the NWR. However, livestock movement patterns may be more important determinants of transmission than static animal density alone. The Adamawa plateau is a major cattle-producing region and experiences extensive seasonal transhumance (40), livestock trade, and congregation at grazing and watering points, all of which create opportunities for transmission between herds over large spatial scales. In addition, the VIN Division around Ngaoundere sees many animals passing through to the abattoir and the rail head for transport to the capital, Yaounde. A new road from Ngaoundere to Yaounde is further helping to increase the influx of cattle, potentially increasing mixing that cannot be captured by the data.

It is also possible that there are different serotypes driving different transmission rates. As mentioned earlier SAT2 was circulating in the VIN and A in the NWR. Analysis of the isolates available in 2019 (38) suggest that different topotypes within the different serotypes are evolving and spreading at different rates. In particular, the SAT2 VII had a very high diffusion rate that may help explain the higher R_0_ estimates observed. There are also differences in the ability of the NSP test to detect antibodies to different serotypes with some evidence that SATs, in particular, may be less well detected (41). However, given the higher seroprevalences in the VIN that are more likely to be related to SAT2, this does not seem like a likely explanation for the observed differences. Nevertheless, it is undoubtedly one of the major limitations of the study that we do not have serotype-specific data.

The estimated R₀ values of 2.05 (95% CI:1.93–2.18) for the NWR and 3.04 (95% CI:2.84–3.26) for the VIN fall within the range reported for FMD in other endemic and epidemic settings. A review of published studies reported values ranging from below 1 to over 13, with a median R₀ of approximately 1.7 (42). Experimental studies in cattle have estimated within-pen R₀ values of approximately 4.4 (43), while farm-level transmission during the 2001 United Kingdom epidemic was estimated to be between 3.5 and 4.5 (44). Direct comparison between studies is difficult because estimates depend strongly on scale, setting, host population structure, and methodology. Nevertheless, the estimates generated here are biologically plausible and consistent with sustained endemic transmission.

When adjusted for population immunity and test performance, the corresponding effective reproductive numbers (R_t_) were estimated at 1.23 (95% CI: 1.15–1.30) in the NWR and 1.14 (95% CI: 1.06–1.22) in the VIN. Despite the higher R_0_ in the VIN, its greater level of population immunity resulted in a lower R_t_ than in the NWR, where a larger susceptible fraction sustained comparatively higher ongoing transmission risk.

However, the R₀ estimates also highlight the challenges of achieving control through vaccination alone. The critical vaccination threshold (1–1/R_0_) was estimated at 51% for the NWR and 67% for the VIN. The overall design-adjusted animal-level seroprevalence of FMDV antibodies was 38.2% in the NWR compared to 56.7% in the VIN, indicating that population immunity remained below the critical threshold required to interrupt transmission at both sites, consistent with R_t_ exceeding 1. The shortfall was greater in the NWR (12.8 percentage points below the threshold) than in the VIN (10.3 percentage points below the threshold), suggesting that a comparatively larger gain in population immunity is needed in the NWR to achieve control. Achieving and sustaining these levels of effective immunity would require good vaccine matching, and repeated vaccination campaigns, all of which are challenging under the logistical and financial constraints faced by livestock services in Cameroon.

The risk factor analyses identified several factors associated with FMD seropositivity. In univariable analyses, herd connectivity indicators such as shared watering points and recent cattle purchases were strongly associated with evidence of infection, supporting the importance of contact networks in maintaining transmission. Unexpectedly, transhumance and reported contact with buffalo appeared to be protective in the univariable analysis. These findings are unlikely to reflect true biological protection and were no longer evident after accounting for confounding, particularly by study area. The results therefore suggest that geographical and management differences between the two production systems explain much of the apparent association. Although African buffalo are recognized reservoirs of Southern African Territories (SAT) serotypes in parts of Africa (45, 46), their role in maintaining transmission in Cameroon remains uncertain and warrants further investigation.

Bovine viral diarrhoea virus (BVDV) serostatus was positively associated with FMD seropositivity in the final model. While BVDV-induced immunomodulation could theoretically influence susceptibility to infection, another plausible explanation is that BVDV seropositivity acts as a proxy for management practices and contact structures that increase exposure to multiple infectious diseases. BVDV seropositivity may therefore act as a marker of animals embedded within highly connected production systems characterized by frequent movements, trade, and contact at communal grazing and watering points. These same mechanisms are known to facilitate FMD transmission, suggesting that BVDV status captures the underlying heterogeneity in exposure risk and shared transmission pathways rather than reflecting only a direct biological interaction. In contrast, the association with paratuberculosis was weaker and may reflect residual confounding by age, while enzootic bovine leukosis and bovine tuberculosis serostatus showed no clear relationship with FMD exposure. Although cross-sectional serological and herdsmen survey data cannot establish the temporal sequence of exposure and co-infection events, nor fully capture the complexity of livestock-livestock and livestock-wildlife interactions, the role of shared grazing areas and watering points in facilitating the direct and indirect transmission of FMD and other transboundary animal diseases has been extensively documented (47). Nevertheless, further research is warranted to better characterize these contact structures and transmission pathways in transhumant livestock.

From a disease-control perspective, the results suggest that blanket vaccination programs designed solely to achieve herd immunity thresholds are unlikely to be feasible under current conditions. Instead, risk-based approaches may provide a more practical and cost-effective strategy. Prioritising younger animals before their first major movements, vaccinating ahead of transhumance periods, and targeting areas characterized by intense herd mixing may yield greater impact than uniform vaccination across all herds (48). Strengthening surveillance and ensuring that vaccine strains are antigenically matched to contemporary FMD viruses circulating within Pool 5 should also be priorities. Post-vaccination monitoring, including serological evaluation of vaccine-induced immunity, would provide important evidence to optimize future control programs.

Several limitations should be considered when interpreting these findings. First, the analysis is based on a cross-sectional serological survey and therefore infers transmission dynamics from age-specific patterns rather than directly observing infection events through time. The catalytic models assume that NSP seropositivity reflects cumulative exposure and that antibody persistence is sufficiently long relative to the age structure of the population; while these assumptions are supported by previous studies, deviations could influence FOI estimates. Second, age estimates relied partly on field assessments and may be subject to misclassification, particularly in older animals. Third, the risk factor data were not originally collected specifically for FMD investigations and therefore lacked detail on several potentially important epidemiological variables linked to viral transmission, including movement frequency, market attendance, and herd-level biosecurity practices. However, we do know that at the time of the study there was no FMD vaccination program and farmers could not access FMD vaccines in Cameroon. Finally, the study was conducted in two regions during a single time period and may not fully capture temporal variation in transmission intensity or be representative of all cattle production systems in Cameroon. Despite these limitations, the consistency between the serological patterns, force-of-infection estimates, and epidemiological observations provides confidence that the findings capture important features of FMD transmission in endemic cattle populations.

An important consideration in interpreting these results is the distinction between age-specific catalytic models and time-varying force-of-infection approaches. The present analysis, like most NSP-based serological studies in endemic settings (39), infers FOI from cross-sectional data stratified by age under the assumptions of stationary viral transmission over time, homogeneous mixing within age strata, and constant mortality/life expectancy. NSP serology also reflects cumulative exposure rather than recent infection or protection, and antibody persistence may vary between individuals, potentially smoothing temporal signal and biasing FOI estimates toward lower variability. NSP antibodies measures the structural protein obtained during infection and there is no correlation with neutralising antibodies and protection. These assumptions may not fully reflect the structured nature of pastoral and agropastoral systems in the study areas nor account for the influence of externalities on local disease dynamics. Therefore, the FOI estimates presented here represent a temporal average across periods of higher and lower transmission intensity over the life course of the sampled cohort rather than a stable underlying rate. In contrast, longitudinal studies, such as those described by Gubbins et al. (49) in Nigeria and Pomeroy *et al*. in Cameroon (39), allow FOI to vary explicitly through time and can capture epidemic pulses, seasonal forcing, or changes in control pressure such as post-outbreak interventions or mass vaccination campaigns. Similarly, time-varying approaches such as those discussed by Sevidzem et al. (50) highlight that FOI in FMD endemic regions is unlikely to be constant, particularly in settings characterized by episodic movements, transhumance, and heterogeneous herd connectivity (40, 51) while also highlighting serotype-specific differences in FOI and Rt. Taken together, these limitations indicate that while the results provide robust evidence of substantial and spatially heterogeneous FMD transmission at the time of data collection in 2013, they should be interpreted as an integrated measure of lifetime transmission intensity rather than real-time transmission dynamics and are best complemented by longitudinal serological follow-up or outbreak-based transmission studies. Studying FMD in these endemic settings, where different serotypes are circulating, is an under-utilized approach and there is enormous potential to improve our understanding of FMD transmission, evolution and risk to FMD-free countries by working with colleagues in endemic settings.

## Conclusion

This study provides field-based estimates of FMD transmission dynamics in two contrasting cattle production systems in Cameroon, using age-stratified NSP seroprevalence to derive the force of infection, R_0_, and R_t_. Both sites showed evidence of substantial, endemic, lifelong transmission, with the Vina Division consistently demonstrating higher exposure intensity than the Northwest Region across every metric measured — seroprevalence, force of infection, and R_0_. Critically, however, this pattern reversed for R_t_: greater existing population immunity in the VIN reduced its effective reproductive number below that of the NWR, despite the VIN’s higher estimated transmission intensity. This finding illustrates why control planning cannot rely on R_0_ alone, and why estimates of current population immunity are essential for interpreting ongoing transmission risk and targeting interventions.

## Data Availability

The datasets presented in this study can be found in online repositories at https://doi.org/10.7488/ds/8184.
