# Supplementary material for: Sero-prevalence, risk factor and force of infection estimates for foot-and-mouth disease in two cattle rearing areas of Cameroon
Source: Front Vet Sci. 2026 Oct 22;13:1959956. doi: 10.3389/fvets.2026.1959956 (PMC13735110; doi:10.3389/fvets.2026.1959956)
Supplement: Supplementary file 1 [file Supplementary_file_1.docx]

**Sero-prevalence, risk factor and force of infection estimates for foot-and-mouth disease in two cattle rearing areas of Cameroon**

Barend M.deC. Bronsvoort^1^ *, Georgina Limon^2^ , Lucy Ndip^3^ , Vincent N. Tanya^4^, Kenton L. Morgan5 , Victor Ngu Ngwa^6^, Charles K. Nfon^7^ , Robert F. Kelly^1^ , Bram A. D. van Bunnik^1^ and Lina González Gordon^1^ 

1 Epidemiology, Economics and Risk Assessment (EERA) Group, The Roslin Institute, Royal (Dick) School of Veterinary Studies, University of Edinburgh, Midlothian, United Kingdom, 
2 The Pirbright Institute, Surrey, United Kingdom, 
3 Laboratory of Emerging Infectious Diseases, University of Buea, Buea, Cameroon, 
4 Cameroon Academy of Sciences, Yaoundé, Cameroon, 
5 Institute of Veterinary Sclence, University of Liverpool, Liverpool, United Kingdom, 
6 School of Veterinary Medicine and Sciences, University of Ngaoundere, Ngaoundere, Cameroon, 
7 National Centre for Foreign Animal Disease, Winnipeg, MB, Canada

*mark.bronsvoort@ed.ac.uk

**Supplementary Material – Appendix A**

Bayesian No Gold Standard Estimate of the FMD ELISA after adjusting the cut-off to 40%.

Script for running in R.

#Test 1 FMD50

#Test 2 FMD40

Test order --/-+/+-/++

```
d <- matrix(c(453,0,15,282,305,0,21,422),2,4)

n <- c(750,748)

dd1<-list( n.pop2=2, n=n,  d=d)


modelData <- dump.format(dd1)
modelInit1 <- dump.format(list(SeFMD50=0.5, SpFMD50=0.95, SeFMD40= 0.67, SpFMD40=0.98,p=runif(length(n), 0.1, 0.3)))
modelInit2 <- dump.format(list(SeFMD50=0.4, SpFMD50=0.93, SeFMD40= 0.57, SpFMD40=0.97,p=runif(length(n), 0.1, 0.3)))
modelInit3 <- dump.format(list(SeFMD50=0.7, SpFMD50=0.90, SeFMD40= 0.47, SpFMD40=0.96,p=runif(length(n), 0.1, 0.3)))
modelInits <- c(modelInit1, modelInit2,  modelInit3)

# ====================================
# =  define model string for runjags =
# ====================================

modelString <- "
model{

	for (j in 1:n.pop2){
  d[j, 1:4] ~ dmulti(peg[j, 1:4], n[j])
  p[j] ~ dunif(0, 1)
  peg[j,4] <- p[j]*(SeFMD50*SeFMD40+covDp) + (1-p[j])*((1-SpFMD50)*(1-SpFMD40)+covDn)
  peg[j,2] <- p[j]*(SeFMD50*(1-SeFMD40)-covDp) + (1-p[j])*((1-SpFMD50)*SpFMD40-covDn)
  peg[j,3] <- p[j]*((1-SeFMD50)*SeFMD40-covDp) + (1-p[j])*(SpFMD50*(1-SpFMD40)-covDn)
  peg[j,1] <- p[j]*((1-SeFMD50)*(1-SeFMD40)+covDp) + (1-p[j])*(SpFMD50*SpFMD40+covDn)
  }

  ls <- (SeFMD50-1)*(1-SeFMD40)
  us <- min(SeFMD50,SeFMD40) - SeFMD50*SeFMD40
  lc <- (SpFMD50-1)*(1-SpFMD40)
  uc <- min(SpFMD50,SpFMD40) - SpFMD50*SpFMD40
  rhoD <- covDp / sqrt(SeFMD50*(1-SeFMD50)*SeFMD40*(1-SeFMD40))
  rhoDc <- covDn / sqrt(SpFMD50*(1-SpFMD50)*SpFMD40*(1-SpFMD40))


  SeFMD50 ~ dbeta(94,6)
  SpFMD50 ~ dbeta(99,1)
  SeFMD40 ~ dbeta(15,2)
  SpFMD40 ~ dbeta(15,1)
  covDn ~ dunif(lc, uc)
  covDp ~ dunif(ls, us)
  # covDn <- 0
  # covDp <- 0
}
"

print(system.time(
  x <- run.jags(	model = modelString,
                 monitor = c("SeFMD50", "SpFMD50", "SeFMD40", "SpFMD40", "covDn", "covDp", "p"),
                 data = modelData,
                 burnin = 20000,
                 sample = 300000,
                 thin = 1,
                 n.chains=3,
                 inits = modelInits
  )
))


x <- list(	result = as.mcmc.list(x[[1]]),
           model = modelString,
           data = modelData)


print(summary(x[[1]]))
```

Results

```
1. Empirical mean and standard deviation for each variable,
   plus standard error of the mean:

            Mean      SD  Naive SE Time-series SE
SeFMD50 0.940023 0.01828 1.927e-05      0.0003710
SpFMD50 0.995983 0.00553 5.829e-06      0.0001539
SeFMD40 0.973027 0.01842 1.942e-05      0.0008406
SpFMD40 0.981374 0.01248 1.316e-05      0.0001042
covDn   0.003118 0.00507 5.345e-06      0.0001432
covDp   0.023778 0.01664 1.754e-05      0.0007489
p[1]    0.397012 0.02032 2.142e-05      0.0001310
p[2]    0.599338 0.02206 2.325e-05      0.0002212

2. Quantiles for each variable:

             2.5%       25%      50%      75%   97.5%
SeFMD50 8.983e-01 0.9293000 0.942233 0.952913 0.97003
SpFMD50 9.812e-01 0.9950450 0.997837 0.999169 0.99993
SeFMD40 9.290e-01 0.9622550 0.976393 0.987412 0.99770
SpFMD40 9.524e-01 0.9739190 0.983486 0.991071 0.99866
covDn   9.458e-06 0.0003262 0.001237 0.003711 0.01691
covDp   1.107e-03 0.0106252 0.020927 0.033858 0.06290
p[1]    3.580e-01 0.3832250 0.396770 0.410514 0.43760
p[2]    5.574e-01 0.5843830 0.598845 0.613716 0.64426
```

**Supplementary Material – Appendix B**

**Sensitivity analysis of R_0_/R_t_ to life expectancy**

```
calc_95perc_CI <- function(cambtb_fmd, L){
  VIN <- cambtb_fmd |> filter(strata1 == "VIN")
  x <- VIN
  # VINA Rt estimation
  mu<-1/L   #1/mu is average life expectancy
  agep <- x %>% subset(., strata1 =="VIN") %>% filter(FMD_ABC_PN_ELISA_40 == 1) %>% dplyr::select(ANIAGE)
  agep <- agep$ANIAGE
  agen <- x %>% subset(., strata1 =="VIN") %>% filter(FMD_ABC_PN_ELISA_40 == 0) %>% dplyr::select(ANIAGE)
  agen <- agen$ANIAGE

  # 95% interval for R0:
  # Use a (log) likelihood ratio test,
  # lambda_LR = -2[LL(theta_0) - LL(theta_bar)]
  # LL(theta_0) is the maximum of LLR0, LL(theta_bar) is the LL for the varying value of R0.
  # find R0 where lambda_LR is equal to critical value of chi-square distribution with 1 df
  # (difference in dimensionality between theta_0 and theta_bar)

  # Find maximum LLRt:
  LLR0_fun <- function(R0, mu, an, ap){
    sum(log(exp(-1*an*mu*(R0-1)))) + sum(log(1-exp(-1*ap*mu*(R0-1))))
  }

  maxLL_VIN = optimise(f=LLR0_fun, c(1,5), maximum = TRUE, mu, an=agen, ap=agep)$maximum

  #R is really bad at solving functions etc. so we have to  add in the critical value already here...:
  #Crititcal value of chisqaure distribution for p=0.05, df=1:
  crit_value = qchisq(1-0.05,1)

  lambda_LR <- function(R01, mu1, an1,ap1){
    -2*(LLR0_fun(maxLL_VIN, mu1,an1,ap1) - LLR0_fun(R01, mu1, an1, ap1)) + crit_value
  }

  lower_root_VIN = uniroot(lambda_LR, c(2,maxLL_VIN), mu1=mu, an1=agen, ap1=agep)$root
  upper_root_VIN = uniroot(lambda_LR, c(maxLL_VIN,7), mu1=mu, an1=agen, ap1=agep)$root


  NWR <- cambtb_fmd |> filter(strata1=="NWR")
  x <- NWR
  mu <- 1/L   #1/mu is average life expectancy
  agep <- x %>% subset(., strata1 =="NWR") %>% filter(FMD_ABC_PN_ELISA_40 == 1) %>% dplyr::select(ANIAGE)
  agep <- agep$ANIAGE
  agen <- x %>% subset(., strata1 =="NWR") %>% filter(FMD_ABC_PN_ELISA_40 == 0) %>% dplyr::select(ANIAGE)
  agen <- agen$ANIAGE

  # 95% interval for R0:
  # Use a (log) likelihood ratio test,
  # lambda_LR = -2[LL(theta_0) - LL(theta_bar)]
  # LL(theta_0) is the maximum of LLR0, LL(theta_bar) is the LL for the varying value of R0.
  # find R0 where lambda_LR is eqaul to critical value of chi-square distribution with 1 df
  # (difference in dimensionality between theta_0 and theta_bar)

  # Find maximum LLRt:
  LLR0_fun <- function(R0, mu, an, ap){
    sum(log(exp(-1*an*mu*(R0-1)))) + sum(log(1-exp(-1*ap*mu*(R0-1))))
  }

  maxLL_NWR = optimise(f=LLR0_fun, c(1,5), maximum = TRUE, mu, an=agen, ap=agep)$maximum

  #R is really bad at solving functions etc. so we have to  add in the critical value already here...:
  #Crititcal value of chisqaure distribution for p=0.05, df=1:
  crit_value = qchisq(1-0.05,1)

  lambda_LR <- function(R01, mu1, an1,ap1){
    -2*(LLR0_fun(maxLL_NWR, mu1,an1,ap1) - LLR0_fun(R01, mu1, an1, ap1)) + crit_value
  }

  lower_root_NWR = uniroot(lambda_LR, c(1.5,maxLL_NWR), mu1=mu, an1=agen, ap1=agep)$root
  upper_root_NWR = uniroot(lambda_LR, c(maxLL_NWR,5), mu1=mu, an1=agen, ap1=agep)$root

  output = tibble("site" = c("VIN", "NWR"),
                  "est" = c(maxLL_VIN, maxLL_NWR),
                  "lower_bound" = c(lower_root_VIN, lower_root_NWR),
                  "upper_bound" = c(upper_root_VIN, upper_root_NWR))
  return(output)
}


cambtb_fmd = read_csv("cambtb_fmd_datastore_20260904.csv")

L_values <- c(4,5,6,7,8)

results <- map(L_values, ~ calc_95perc_CI(cambtb_fmd, .x))

plot_data <- map2_dfr(
  results,
  L_values,
  ~ mutate(.x, L = .y)
)

ggplot(plot_data, aes(x = L, y = est, colour = site)) +
  geom_line() +
  geom_point() +
  geom_errorbar(
    aes(ymin = lower_bound, ymax = upper_bound),
    width = 0.1
  ) +
  labs(x="Life expectancy", y=expression(R[0]), colour="Site") +
  theme_minimal()

seroprev_div_region <- tibble(region=c(rep("NWR",7), rep("VIN", 6)),
                              division=c("Boyo","Bui","Donga Mantung","Menchum",
                                         "Mezam","Mono","Ngoketunjia","Belel",
                                         "Martap","Mbé","Ngan-ha","Ngaoundere",
                                         "Nyambaka"),
                              seroprev = c(0.37,0.51,0.26,0.2,0.38,0.32,0.78,0.64,0.58,0.8,0.37,0.79,0.51)
                              )

sens = 0.973
spec = 0.981

mean_seroprev = seroprev_div_region |>
  group_by(region) |>
  summarise(mean_seroprev=mean(seroprev))

Rt = left_join(plot_data, mean_seroprev, by=c("site"="region"))
Rt <- Rt |>
  mutate(unadj_Rt = est*(1-mean_seroprev),
         adj_prev = (mean_seroprev+(spec-1))/(sens+spec-1),
         adj_Rt = est * (1-adj_prev),
         adj_LB = lower_bound * (1- adj_prev),
         adj_UB = upper_bound * (1 - adj_prev)
         )
```

| site | Life Expect | R0 | R0 LB | R0 UB | Mean  seroprev | unadj_Rt | adj_prev | adj_Rt | adj_LB | adj_UB |
| --- | --- | --- | --- | --- | --- | --- | --- | --- | --- | --- |
| VIN | 4 | 2.36 | 2.22 | 2.51 | 0.62 | 0.91 | 0.62 | 0.89 | 0.83 | 0.94 |
| NWR | 4 | 1.70 | 1.62 | 1.79 | 0.40 | 1.02 | 0.40 | 1.02 | 0.97 | 1.07 |
| VIN | 5 | 2.70 | 2.53 | 2.88 | 0.62 | 1.04 | 0.62 | 1.01 | 0.95 | 1.08 |
| NWR | 5 | 1.88 | 1.78 | 1.98 | 0.40 | 1.12 | 0.40 | 1.12 | 1.06 | 1.19 |
| VIN | 6 | 3.04 | 2.84 | 3.26 | 0.62 | 1.17 | 0.62 | 1.14 | 1.06 | 1.22 |
| NWR | 6 | 2.05 | 1.93 | 2.18 | 0.40 | 1.22 | 0.40 | 1.23 | 1.15 | 1.30 |
| VIN | 7 | 3.38 | 3.14 | 3.64 | 0.62 | 1.30 | 0.62 | 1.27 | 1.18 | 1.37 |
| NWR | 7 | 2.23 | 2.09 | 2.38 | 0.40 | 1.33 | 0.40 | 1.33 | 1.25 | 1.42 |
| VIN | 8 | 3.72 | 3.45 | 4.02 | 0.62 | 1.43 | 0.62 | 1.40 | 1.29 | 1.51 |
| NWR | 8 | 2.40 | 2.24 | 2.57 | 0.40 | 1.43 | 0.40 | 1.44 | 1.34 | 1.54 |
